# Single-Cell RNA Sequencing Revealed the Heterogeneity of Gonadal Primordial Germ Cells in Zebra Finch (*Taeniopygia guttata*)

**DOI:** 10.3389/fcell.2021.791335

**Published:** 2021-12-09

**Authors:** Kyung Min Jung, Minseok Seo, Young Min Kim, Jin Lee Kim, Jae Yong Han

**Affiliations:** ^1^ Department of Agricultural Biotechnology and Research Institute of Agriculture and Life Sciences, College of Agriculture and Life Sciences, Seoul National University, Seoul, South Korea; ^2^ Department of Computer Convergence Software, Korea University, Sejong, South Korea

**Keywords:** zebra finch (*Taeniopygia guttata*), embryonic gonad, primordial germ cell, single-cell RNA sequencing, heterogeneity

## Abstract

Primordial germ cells (PGCs) are undifferentiated gametes with heterogeneity, an evolutionarily conserved characteristic across various organisms. Although dynamic selection at the level of early germ cell populations is an important biological feature linked to fertility, the heterogeneity of PGCs in avian species has not been characterized. In this study, we sought to evaluate PGC heterogeneity in zebra finch using a single-cell RNA sequencing (scRNA-seq) approach. Using scRNA-seq of embryonic gonadal cells from male and female zebra finches at Hamburger and Hamilton (HH) stage 28, we annotated nine cell types from 20 cell clusters. We found that PGCs previously considered a single population can be separated into three subtypes showing differences in apoptosis, proliferation, and other biological processes. The three PGC subtypes were specifically enriched for genes showing expression patterns related to germness or pluripotency, suggesting functional differences in PGCs according to the three subtypes. Additionally, we discovered a novel biomarker, *SMC1B*, for gonadal PGCs in zebra finch. The results provide the first evidence of substantial heterogeneity in PGCs previously considered a single population in birds. This discovery expands our understanding of PGCs to avian species, and provides a basis for further research.

## Introduction

The heterogeneity of primordial germ cells (PGCs), the only cells capable of transferring genetic information to the next generation, during early embryonic development is conserved across a wide range of taxa (Han, 2009; Nguyen et al., 2019). Initial PGCs are heterogeneous with respect to their contribution to germline development (Ueno et al., 2009). In zebrafish (*Danio rerio*), chemokine responsiveness and motility, which determine migration toward gonads during early development, differ among individual cells. PGCs showing aberrant migration are eliminated by apoptosis (Koprunner et al., 2001; Weidinger et al., 2003; Reichman-Fried et al., 2004). In mice (*Mus musculus*), PGCs show asynchronous migration, in which some cells lag behind or move to an ectopic location via niche signals or intrinsic heterogeneity; aberrant migration is closely related to cell survival (Molyneaux et al., 2001; Laird et al., 2011; Cantú et al., 2016). The heterogeneity of PGCs can also be caused by differences in proliferation, as observed in flies (*Drosophila melanogaster*). Highly proliferative PGC populations are overrepresented in the germline and may compete for access to signaling factors essential for development (Gilboa and Lehmann, 2006). PGCs with the potential to become germ cell tumors and harboring deleterious mutations can be eliminated, and only a subset of the initial population completes fetal development (Runyan et al., 2008).

Morphological analyses of germ cell heterogeneity and genetic manipulation of germ cells have been accelerated by development of single-cell RNA sequencing (scRNA-seq) techniques, which enable transcriptome studies and detailed lineage specification. ScRNA-seq in human fetal germ cells has revealed transcriptional and epigenetic heterogeneity as well as variation in susceptibility to apoptosis (Reznik et al., 2019; Nguyen et al., 2020). Heterogeneity and developmental asynchrony in female germ cell populations has also been identified in mice (Zhao et al., 2020). In avian species, scRNA-seq was used to evaluate chicken embryonic gonadal cells at several developmental stages; however, this analysis was focused on gonadal sex determination (Estermann et al., 2020). Although the selection process acting on early germ cells is linked to germ cell quality and fertility, studies of the heterogeneity of avian germ cells are lacking.

The zebra finch (*Taeniopygia guttata*) is a representative model bird in evolutionary and neurobiological studies, and assembly of its complete genome sequence has opened up new and interesting avenues for related research fields (Mello, 2014). Zebra finch PGCs have distinct migratory and numerical characteristics at early developmental stage from those of other avian species. And the zebra finch is used for basic research and germline transgenics, indicating the importance of further germ cell research (Jung et al., 2019; Gessara et al., 2021). In zebra finch, PGCs are widely distributed in the early embryo, complete their migration to the germinal crescent at Hamburger Hamilton stage (HH) 6 and enter the circulation through the anterior vitelline vein at HH stage 10. After settlement of circulating PGCs into the genital ridge, PGCs are maintained prior to sex differentiation. The well-known chicken PGCs undergo sexual differentiation from HH stage 30, and the zebra finch also showed a clear bilateral gonad asymmetry from HH stage 30, so HH stage 28 could be chosen to study undifferentiated gonad PGCs (Jung et al., 2019; Estermann et al., 2020).

In this study, we performed a scRNA-seq analysis of zebra finch embryonic gonadal cells at HH stage 28 to evaluate the heterogeneity of PGCs. We identified three distinct subpopulations of gonadal PGCs in a single developmental stage. These results provide interesting insights into zebra finch PGCs, including the first evidence for a cellular level selective event during embryonic development in avian species.

## Materials and Methods

### Experimental Animals and Animal Care

The experimental use of zebra finches was approved by Institutional Animal Care and Use Committee of Seoul National University, Korea (IACUC, SNU-200305-2-1). All methods were carried out in accordance with ARRIVE (Animal Research: Reporting of *In Vivo* Experiments) guidelines and the standard operating protocols of Animal Genetic Engineering Laboratory at Seoul National University.

### Gonad Sample Preparation

Gonads were collected from the abdomens of zebra finch embryos at Hamburger and Hamilton (HH) stages 26, 28, 34, 40, and on hatching day (Hamburger and Hamilton, 1951). The embryonic stages were identified according to incubation time and developmental features (Murray et al., 2013). Eggs were opened 3 days prior to sampling HH stage 28 gonads, and 1 µl of blood was taken for molecular sexing. In particular, the chromodomain-helicase-DNA binding protein (CHD) 1 gene was amplified by PCR using genomic DNA extracted from blood with previously described primers (Soderstrom et al., 2007). The gonads of sex-identified individuals were used for scRNA-seq.

### Single-Cell Library Preparation and Sequencing

Seven male and seven female embryonic gonads at HH stage 28 were collected for each sex, pooled in 100 µl of trypsin (0.05%), and incubated at 37°C for 5 min with gentle pipetting. Digestion was stopped by addition of 100 µl of Dulbecco’s Modified Eagle’s Medium (Hyclone, Logan, UT, United States) containing 10% fetal bovine serum (Hyclone), followed by washing with 500 µl of PBS. Cells were pelleted down and resuspended in PBS. Single-cell libraries were prepared using the Chromium controller according to the 10× Chromium Next GEM Single Cell 3′ v3.1 protocol (10× genomics document CG000204 Rev D) (10× Genomics, Pleasanton, CA, United States) by Macrogen (Seoul, Korea). Briefly, cell suspensions were diluted in nuclease-free water to achieve a targeted cell count of 10,000. The cell suspension was mixed with master mix and loaded with Single Cell 3′ v3.1 Gel Beads and Partitioning Oil into a Chromium Next GEM Chip G apparatus. RNA transcripts from single-cells were uniquely barcoded and reverse-transcribed within droplets. Next, cDNA molecules were pooled and then was subjected to an end repair process, addition of a single ‘A’ base, and ligation of the adapters. The products were then purified and enriched by PCR to create the final cDNA library. Samples were subjected to 11 cycles of PCR for cDNA amplification and 16 cycles for library amplification. The purified libraries were quantified by qPCR according to the qPCR Quantification Protocol Guide (Kapa Biosystems, Wilmington, MA, United States), and quality was determined using the Agilent Technologies 4200 TapeStation (Santa Clara, CA United States). Then, the libraries were sequenced using the Illumina HiSeq X ten platform (Illumina, San Diego, CA, United States), according to the read length [R1(28) - I1(8) -R2(91)] in the user guide. The number of sequenced reads per library was 720,503,190 in male, and 785,997,522 in female.

### Single-Cell Gene Expression Analysis

Pre-processing and quality control of single-cell sequencing raw data was performed by Macrogene Inc. (Seoul, Korea) based on their in-house pipeline. Briefly, single-cell raw sequencing data were preprocessed using Cell Ranger v5.0.0 (10× Genomics). The raw BCL files from the Illumina HiSeq platform were demultiplexed to generate FASTQ files using “cellranger mkfastq.” Then, these raw FASTQ files were analyzed using “cellranger count” based on the zebra finch reference genome (bTaeGut1_v1) to quantify mapped reads at each cell barcodes. Quantified raw count matrices were analyzed using Seurat 3.1.3. Raw counts for genes expressed in ≥3 cells, and cells with at least 200 detected genes were used for downstream analyses. The percent_mt filtering was omitted because the mitochondrial genes were not annotated in the zebra finch Ensembl database. Dimensionality reduction was performed using principal component analysis (PCA) for identification of highly variable genes. To fine the graph-based clusters, K-nearest neighbor (KNN) graph based on the PCA space were performed with Louvain algorithm. UMAP algorithm (McIntyre et al., 2008) was used to visualize cell-type specific expression patterns. A statistical hypothesis test for detecting genes exhibiting specific expression patterns for each cluster was performed using Wilcox rank sum test with min.pct = 0.25 and logfc.threshold = 0.25. Statistical hypothesis testing of the aggregated count of the three identified germ-cell clusters were performed edgeR. In this study, an FDR-adjusted *p*-value of 0.05 or less after adjustment using the Benjamini & Hochberg method (Benjamini and Hochberg, 1995) was used as the significance cutoff. Gene-Enrichment and Functional Annotation analysis for significant probe list was performed using g:Profiler (https://biit.cs.ut.ee/gprofiler/). Single-cell trajectory reconstruction was performed by Monocle3, which results in the definition of a pseudo-time measurement across root sample group through which the dynamics of gene expression can be examined. Cells were clustered with the Louvain algorithm for community detection and then detected communities were separated into partitions by the pruned kNN algorithm.

### TUNEL Assay

Apoptotic cells were detected by TUNEL assay using TMR red labeling *in situ* cell death detection kit (Roche, Basel, Switzerland) following the manufacturer’s instructions. Whole gonadal cells at HH stage 28 were smeared and fixed onto a glass slide with 4% paraformaldehyde at room temperature for 1 h. Cells were then permeabilized using 0.1% triton X-100 on ice for 2 min. And the cells were then incubated with the TUNEL reaction mixture which labels DNA strand breaks, by terminal deoxynucleotidyl transferee (TdT), which catalyzes polymerization of labelled nucleotides to free 3’-OH DNA ends in a template-independent manner (TUNEL-reaction). Cells induced DNA damage by treatment with DNase Ⅰ for 10 min were used as a positive control, and the cells treated with TUNEL reaction mixture excluding TdT was used as a negative control. After incubation with the tunnel reaction mixture, cells were mounted with ProLong Gold antifade reagent with DAPI and visualized under a confocal fluorescence microscope (Carl Zeiss GmbH, Oberkocken, Germany).

### RT-PCR and Quantitative RT-PCR

To obtain the PGC and GSC populations, whole gonadal cells were short-term cultured *in vitro* according to previous report (Jung et al., 2019). Briefly, whole gonadal cells dissociated with 0.05% of trypsin-EDTA were resuspended in PGC culture medium and cultured in a CO_2_ incubator maintained at 37°C with 5% CO_2_ and 60–70% relative humidity. Floating PGCs and GSCs attached to the bottom of the plate were harvested, respectively, and passaged at intervals of 4–5 days for 10–20 days, and the cells grown enough to extract RNA were used for analysis. Total RNA samples of PGCs or GSCs were prepared using TRIzol reagent (Thermo Fisher Scientific, Waltham, MA, United States). Total RNA samples were then reverse-transcribed into cDNA using the SuperScript III Reverse Transcription Kit (Thermo Fisher Scientific), according to the manufacturer’s protocol. The cDNAs were amplified by PCR using primer sets specific for predicted zebra finch *DAZL*, *DDX4*, *DND1*, *PIWIL1*, *RNF17*, *SMC1B*, *RBM46*, and *HORMAD1* sequences. Glyceraldehyde 3-phosphate dehydrogenase (*GAPDH*) was used as a control. PCR conditions were as follows: 35 cycles at 95°C for 30 s, 60°C for 30 s, and 72°C for 1 min. Gene expression was measured using EvaGreen dye (Biotium, Hayward, CA, United States) and a CFX96 Real-Time PCR Detection System (Bio-Rad, Hercules, CA, United States). Relative gene expression was quantified using the 2-^(DDCt)^ value (DCt = Ct of the *target gene*—Ct of *GAPDH*; DDCt = DCt of PGCs - DCt of GSCs). Total RNA samples from different tissues (including the heart, lung, liver, intestine, muscle, testis, and ovary) from adult male and female zebra finch were isolated using TRIzol reagent, and cDNAs were synthesized using the SuperScript III Reverse Transcription Kit. Total RNA samples from embryonic gonads at different developmental stages (including HH 28, 34, 40, and hatching day) were also prepared following the same protocol. Primer information is listed in Supplementary Table S1.

### Immunohistochemistry of Zebra Finch Embryonic Gonads

Embryonic gonads with mesonephric tissues at HH stage 28 were paraffin-embedded and sectioned (thickness, 8 µm). After deparaffinization, sections were washed with 1× PBS and blocked with a blocking buffer (5% goat serum and 1% bovine serum albumin in PBS) for 1 h at room temperature. Sections were then incubated overnight at 4°C with a rabbit anti–SMC1B antibody, HPA001500 (Atlas Antibodies AB, Stockholm, Sweden). After washing with PBS, sections were incubated with anti-rabbit IgG-Alexa Fluor 594 (Thermo Fisher Scientific) for 1 h at room temperature. After washing with PBS, sections were mounted with ProLong Gold antifade reagent with DAPI and visualized under a confocal fluorescence microscope (Carl Zeiss GmbH).

### Whole-Mount *in situ* Hybridization

Hybridization probes for zebra finch *SMC1B* were made from total RNA extracted from embryonic gonads, which was then reverse-transcribed using the SuperScript III First-Strand Synthesis System (Thermo Fisher Scientific). Next, cDNA was amplified using *SMC1B*-specific primers (forward, 5′-CAG CAC CCT AGA CCT TGA CC-3′ and reverse, 5′-AGG ATT CTC CGG GCT AAG GA-3′). The PCR product was cloned into the pGEM-T Easy Vector System (Promega, Madison, WI, United States). After sequence verification by Sanger single-molecule sequencing, recombinant plasmids containing the target genes were amplified using T7-and SP6-specific primers to prepare the template for labeling the hybridization probes. Digoxigenin (DIG)-labeled sense and antisense *SMC1B* hybridization probes were transcribed *in vitro* using a DIG RNA Labeling Kit (Roche). Whole-mount *in situ* hybridization of zebra finch embryonic gonads with mesonephric tissues at HH stage 28 was performed using a standard protocol with an anti-DIG alkaline phosphatase–conjugated antibody and visualization by a 5-bromo-4-chloro-39-indolyphosphate p-toluidine salt and nitro-blue tetrazolium chloride colorimetric reaction. Afterwards, *in situ*–hybridized samples were paraffin-embedded, sectioned (thickness, 8 µm), and evaluated under a microscope (Nikon).

### Statistical Analysis

All of statistical analysis between the PGCs and GSCs was conducted using the Student’s *t*-test with GraphPad Prism statistical software (GraphPad Software, La Jolla, CA, United States).

## Results

### scRNA-Seq of Gonadal Cells in Zebra Finch

**FIGURE 1:**
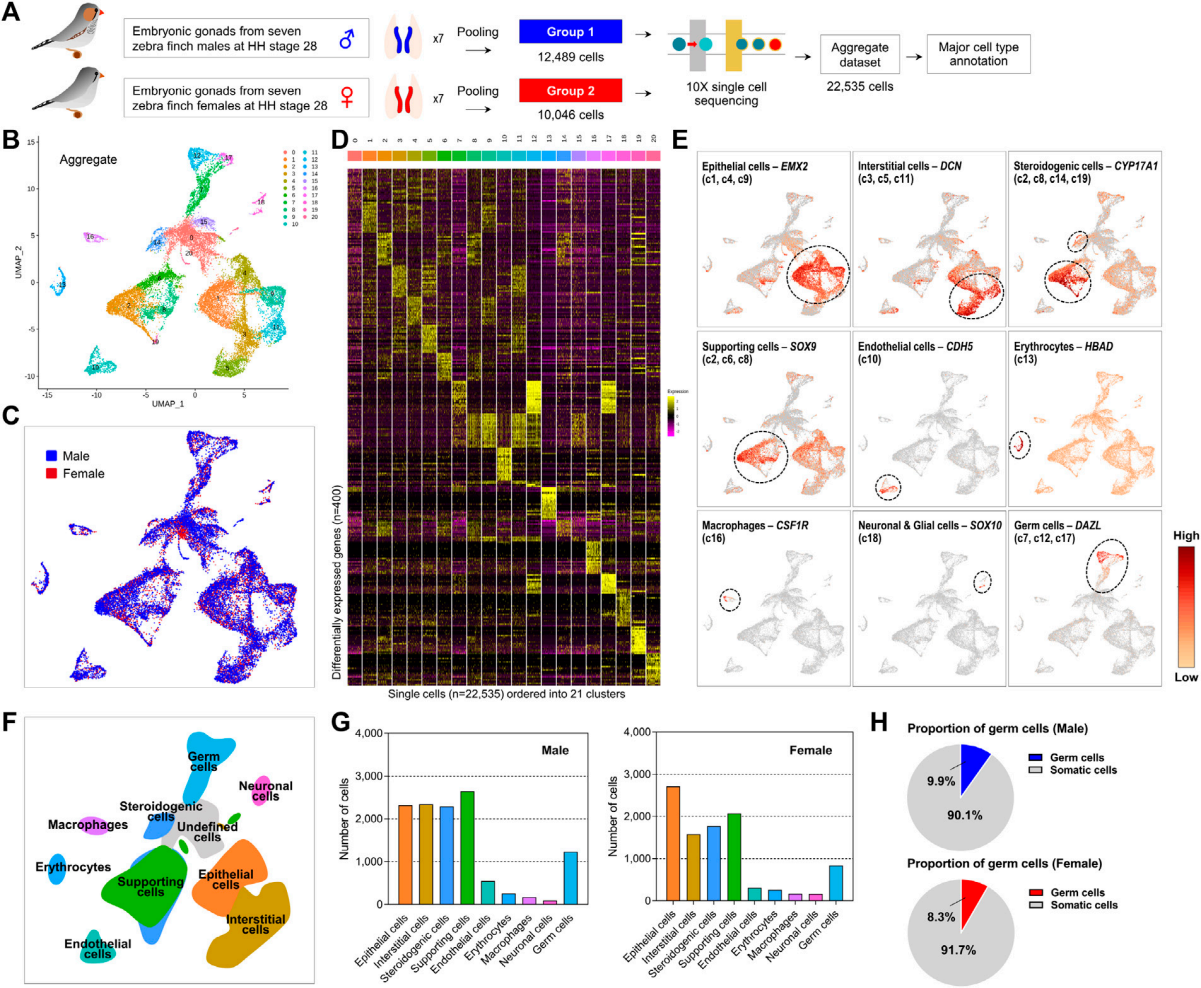
Distinct gonadal cell subpopulations with transcriptional signatures determined by scRNA-seq analysis of zebra finch gonads. **(A)** Schematic processing of dispersed cells from male and female embryonic gonads for single-cell capture and RNA sequencing. **(B)** Aggregated UMAP (Uniform Manifold Approximation and Projection) clustering of all 22,535 cells (12,489 male cells and 10,046 female cells) identified 20 distinct clusters. **(C)** Aggregated UMAP for both sexes show high overlap between the clusters. **(D)** Heatmap of top 20 differentially expressed genes (DEGs) between 20 clusters of zebra finch gonadal cells. **(E)** UMAPs showing expression levels of germ cell and somatic cell marker genes. **(F)** UMAP-based illustration showing nine gonadal cell types. **(G)** Cell counts for nine different cell types. **(H)** Proportion of germ cells in the male and female gonadal cells.

To investigate the undifferentiated gonadal PGCs, we collected whole gonadal cells from zebra finch embryos of known sex at HH stage 28 (Supplementary Figure S1A). Single-cell suspensions of male and female embryonic gonads were captured individually and processed using the 10× Chromium system. After filtering out low quality cells based on the number of genes, and UMIs, in total 12,489 and 10,046 cells were sequenced from pooled male and female gonads (*n* = 7 each), respectively (Figure 1A; Supplementary Figure S1).We clustered cells based on gene expression levels in 22,535 cells, including male and female subjects. We obtained 20 clusters (referred to as c1–c20) using the uniform manifold approximation and projection (UMAP) algorithm (Figure 1B). Cells from both sexes are present in all the clusters, except for cluster 20 (c20), which showed only male-specific expression patterns (Figure 1C; Supplementary Figure S2A). On average, in clusters c1 to c17, the proportion of cells derived from males was relatively high, whereas c15, c18, and c19 showed a relatively high proportion of cells derived from females (Supplementary Figures S2B, S2C). We evaluated 20 cluster-specific expression patterns under the one-vs.-rest comparison strategy, resulting in an average of 454 differentially expressed genes (DEGs) at a 5% significance level after adjustment for multiple comparisons (Figure 1D; Supplementary Figure 2D; Supplementary Table S2). In particular, a number of DEGs (i.e., 1,182 and 1,954 genes) were detected in c12 and c17, respectively, suggesting that these 2 cell clusters are involved in distinct biological processes.

### Identification of Nine Cell Types Based on Cell-Type Specifically Expressed Genes in Zebra Finch Gonads

We characterized 20 cell clusters based on DEGs exhibiting cell-specific expression patterns identified in this study (Figure 1E; Supplementary Figure S3) and previously established cell type classification criteria for chicken species (Hu et al., 2019; Estermann et al., 2020). We successfully annotated nine cell types from gonadal cell clusters (Figure 1F). First, we found that cells assigned to c1, c4, and c9 were epithelial cell populations based on specific upregulation of *KRT18* and *EMX2*. In c3, c5, and c11, *COL3A1*, *COL1A2*, *POSTN*, and *DCN* were specifically expressed, indicating that these populations are interstitial cells. We found that *SOX9* and *DMRT1* were expressed in males, and that *CYP19A1* was specifically expressed in females, in cell clusters c2, c6, and c8, respectively, indicating that these clusters contain supporting cells such as precursors of Sertoli cells or granulosa cells. Furthermore, c2 and c8, along with c14 and c19, expressed the cell type-specific markers *CYP17A1* and *STAR*, representing the steroidogenic cell population. Cell cluster c10 was an endothelial cell population based on specific expression of *CDH5* and *SOX18*, and cells assigned to c13 could be classified as erythrocytes based on expression of several hemoglobin genes such as *HBAD* and *HBAA*. Cell cluster c15 specifically expressed *CDK1* and *UBE2C*, indicating a mitotically active status; however, very few differentially expressed transcripts were unique to c15, making it difficult to assign these cells to a specific type. Levels of macrophage markers *CSF1R*, *LY86*, *SLC40A1*, and *HMOX1* were higher in c16 than in other clusters. In addition, c18, characterized by *SOX10* and *TAGLN3* overexpression, could be considered to be neuronal and glial cells. Interestingly, we observed that PGCs, hitherto known as a single-cell type in avian species, could be subdivided into three groups (c7, c12, and c17) based on cell type-specific gene expression patterns. We confirmed that these clusters commonly expressed germline-specific genes such as *ENSTGUG00000003145* (*DAZL*), *DDX4*, *ENSTGUG00000004779* (*PIWIL1*), and *DND1*, supporting identification of these three clusters as PGCs (Supplementary Table S2).

We found that among these nine cell types, supporting cells, steroidogenic cells, interstitial cells, and epithelial cells accounted for a relatively high proportion of cells in both sexes (Figure 1G). The number of PGCs was similar between sexes, accounting for 9.9% of all sequenced gonadal cells in males and 8.3% in females (Figure 1H).

### Transcriptionally Distinct Three Clusters in Zebra Finch PGCs

**FIGURE 2:**
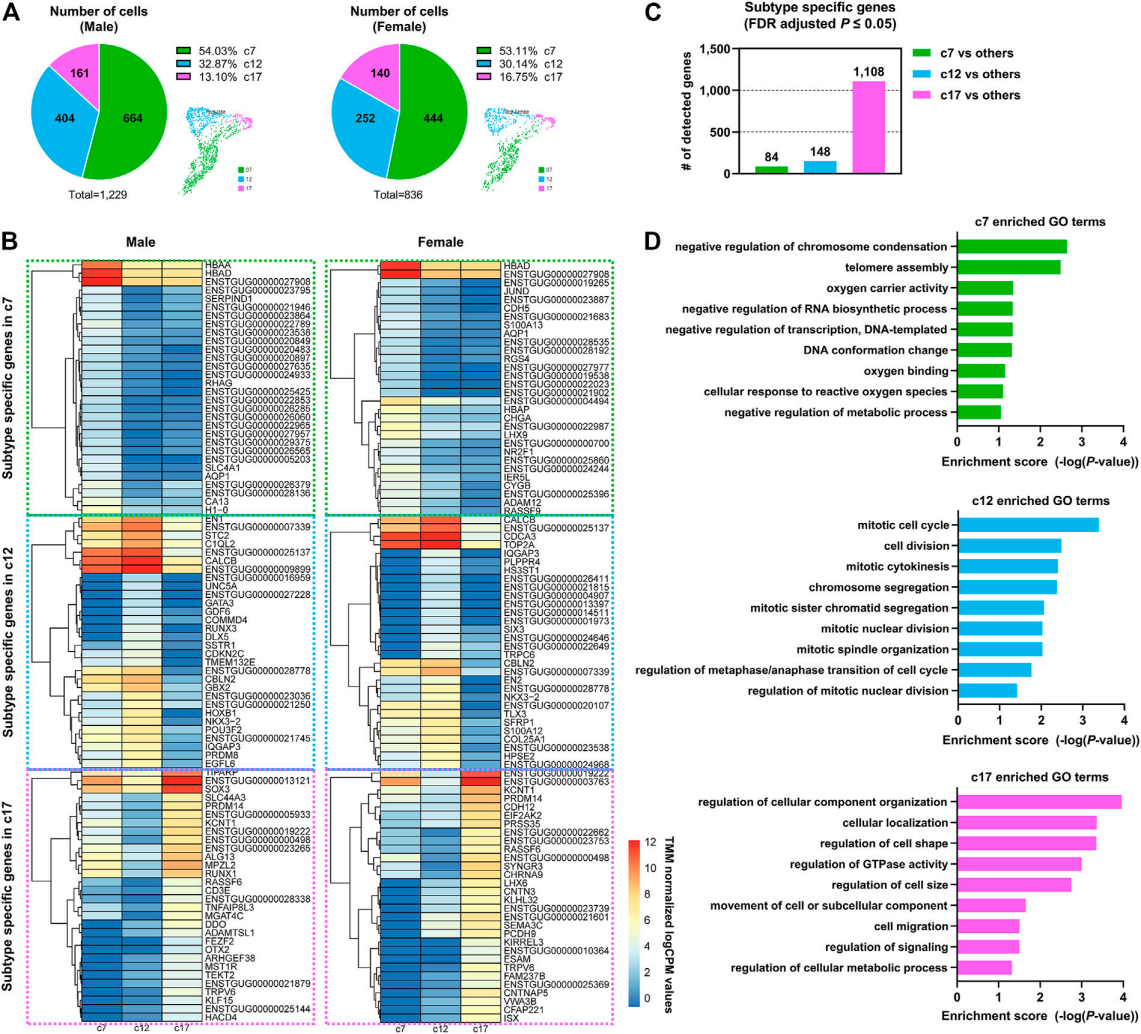
Dynamic gene expression patterns in PGC subpopulations at a single developmental stage. **(A)** The number and portion of PGCs allocated to each subpopulation in both sexes. **(B)** Heatmap of differentially expressed genes (DEGs) between three distinct PGC clusters in both sexes. **(C)** Number of DEGs in one cluster compared to all other clusters. **(D)** Representative Gene Ontology (GO) terms for PGC subpopulation-specific genes.

We further investigated PGC subtypes c7, c12, and c17 to characterize PGC heterogeneity in avian species. Of the cells that were assigned to the PGC clusters, 54.03, 32.87, and 13.10% of male and 53.11, 30.14 and 16.75% of female cells fell into cluster c7, c12, and c17, respectively (Figure 2A), indicating that male and female subjects did not differ in terms of the proportion of each PGC sub-cluster. A comparative expression analysis revealed that 84, 148, and 1,108 genes exhibited subtype-specific expression patterns in c7, c12, and c17, respectively, at a 5% significance level after adjustment for multiple testing (Figure 2C). These genes showing specific expression were upregulated in the particular subtype compared to levels in other subtypes (Figure 2B; Supplementary Table S3).

### Biological Characteristics of Three Subtypes of PGCs Showing Heterogeneity

We performed a functional enrichment analysis of the PGC subtype-specific genes (Figure 2D; Supplementary Table S4) and found that each PGC subpopulation had distinct biological characteristics. Genes related to the GO terms “negative regulation of metabolic process” (e.g., *H3F3A*, *H3F3B*, *NR0B1*, and *BASP1*) and “cellular response to reactive oxygen species” (e.g., *FOS*, *JUN*, *HBAA*, and *HBAD*) were highly expressed in the c7 PGC subtype. In addition, genes involved in *DDIT4*-mediated autophagy were expressed specifically in c7, including the highly expressed *DDIT4* gene, which induce autophagy by blocking mTOR signaling pathways, and *EEF2K* and *AKT1*, expressed at low levels, which are positive regulators of mTOR (Supplementary Tables S3, S4). These features suggest that c7 PGCs undergo cell death, and some apoptotic cells in the gonads were detected by TUNEL assay (Supplementary Figure S4). Mitotic cell cycle-related genes (*NDC80*, *CTBP1*, *PDS5A*, *TOP2A*, *XPO1*, and *KIF20A*) were upregulated in the c12 PGC subtype. The proliferation marker genes *ENSTGUG00000007339* (*BUB1*), *ENSTGUG00000021193* (*MKI67*) and *PLK1* were also more highly expressed in c12 than in other PGC subtypes (Supplementary Tables S3, S4), suggesting that it is a dominant proliferative PGC subtype showing mitotic cell cycle changes. Interestingly, we found that c17 was enriched in many significant GO terms, including GO terms related to the location and composition of the cell, such as “regulation of cellular component organization,” “cellular localization,” “regulation of GTPase activity,” and “cell migration” (Figure 2D; Supplementary Table S4); this indicates that this PGC subtype undergoes diverse biological changes. The detection of enriched mitotic genes in cluster 12 and the various GO terms detected in cluster 17 can be interpreted as distinct PGC populations or as different stages in the life of same population PGCs. The differences in biological pathways revealed by the functional enrichment analysis further demonstrate the heterogeneity of PGCs, which have hitherto been considered to perform the same biological functions.

**FIGURE 3:**
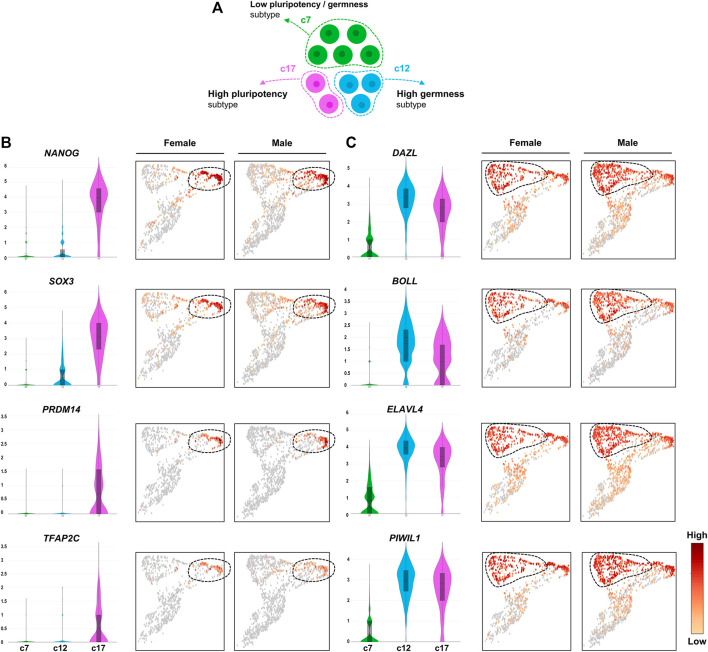
Heterogeneous expression of PGC marker genes. **(A)** Illustration of heterogeneous features among PGC subpopulations. **(B)** UMAPs showing high expression of early PGC marker genes (pluripotency markers) in cluster 17. **(C)** UMAPs showing high expression of late PGC marker genes (germness markers) in cluster 12. The number of the *Y*-axis mean log values.

We wondered whether representative germness- and pluripotency-related genes exhibit specific expression patterns in the newly identified PGC subtypes (Figure 3A). Among well-established pluripotency-related genes in avian species, we found that *ENSTGUG00000013121* (*NANOG*, *P*_adj_: 8.6e-179), *SOX3* (*P*_adj_: 4.06e-173), *PRDM14* (*P*_adj_: 3.83e-160), and *TFAP2C* (Padj: 6.96e-96) were upregulated specifically in c17 (Figure 3B). In the c12 PGC subtype, we observed upregulation of representative germ cell-related genes such as *ENSTGUG00000003145* (*DAZL*, *P*_adj_: 6.79e-47), *BOLL* (*P*_adj_: 1.73e-136), *ELAVL4* (*P*_adj_: 5.43e-07), and *ENSTGUG00000004779* (*PIWIL1*, *P*_adj_: 5.41e-73) (Figure 3C). Although the expression levels of these germness-related genes were highest in c12, relatively high expression levels were also observed in c17. However, in c7, significantly lower levels of such germness- or pluripotency-related genes were observed compared with other subsets, suggesting that the PGC subpopulations have clearly distinct roles in zebra finch development. As a result of additionally performing trajectory analysis for three PGC subtypes, pseudotime was estimated in the order of c7, c17, and c12 (Supplementary Figure S5).

### Novel Markers to Capture PGCs From the Gonads of Zebra Finch

**FIGURE 4:**
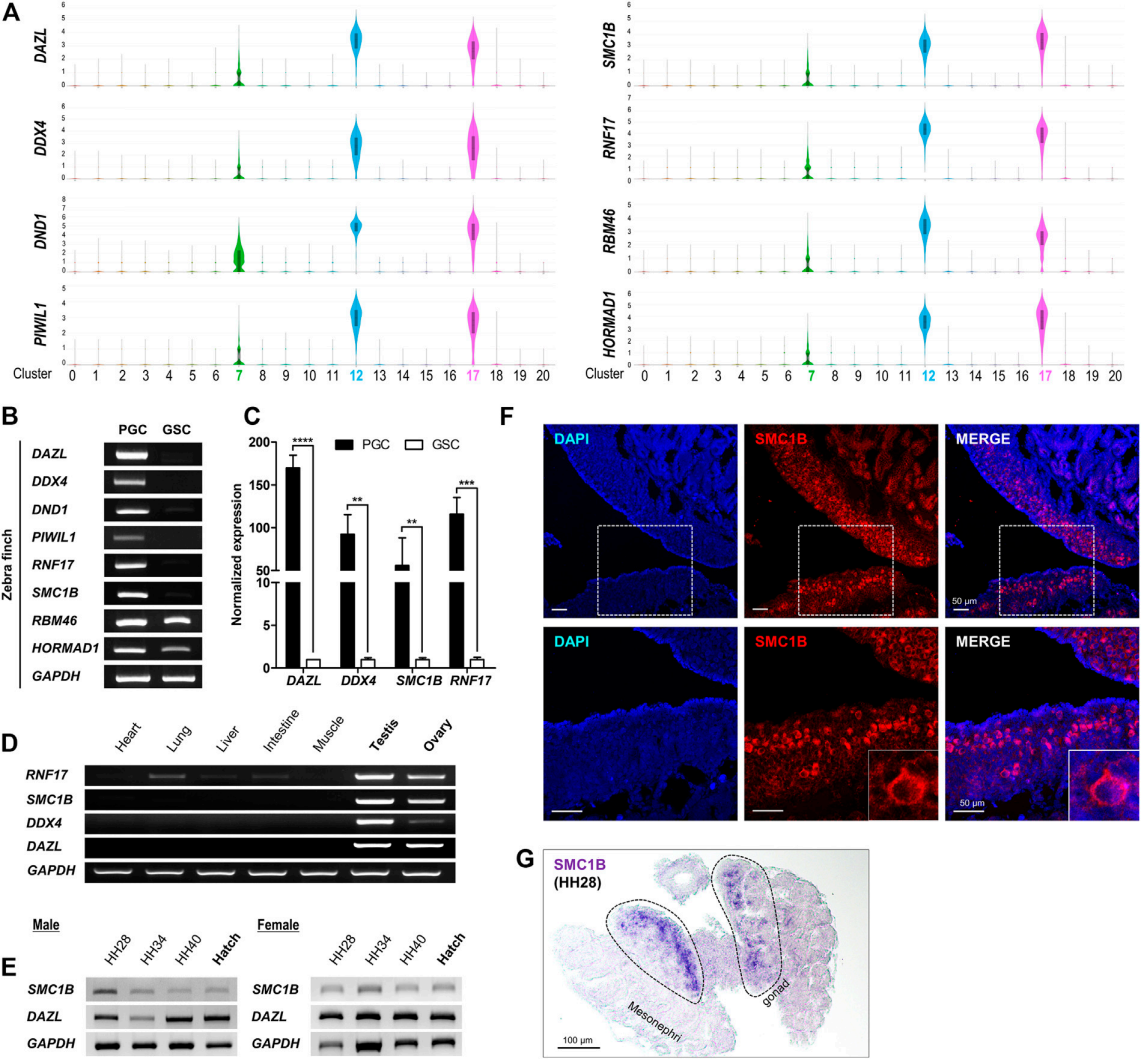
Identification of novel marker genes for zebra finch gonadal PGCs. **(A)** Selected genes from the top 20 DEGs in each PGC cluster. The number of the *Y*-axis mean log values. **(B)** RT-PCR analysis of selected genes in PGCs and GSCs. **(C)** Quantitative RT-PCR analysis of selected genes. Significant differences are shown (Student’s t-test; ****p < 0.0001; ***p < 0.0005; **p < 0.005). **(D)** Tissue-specific expression of selected genes. **(E)** Expression of selected genes according to developmental stage. **(F)** Localization of SMC1B to gonadal cells was analyzed by immunohistochemistry using anti-SMC1B antibodies in zebra finch embryonic gonads at HH stage 28. The dotted boxes in the upper panels are enlarged and shown in the lower panels. **(G)** *In situ* hybridization of *SMC1B* in zebra finch embryonic gonads at HH stage 28.

For chicken or quail, which are representative model species of birds, there are commercially available antibodies such as DAZL and SSEA-1 (Stage-Specific Embryonic Antigen-1) that distinguish PGCs from gonads; however, few antibodies are optimized for zebra finch PGCs, and commercialized antibodies are lacking (Jung et al., 2005; Huss et al., 2019; Ioannidis et al., 2021). Accordingly, it is necessary to discover a marker that is expressed specifically in PGCs. Based on genes expressed specifically in only c7, c12, and c17, we identified PGC-specific genes (Supplementary Figure S6) and selected those that were commonly and highly expressed in PGCs as novel markers (Supplementary Table S5). We demonstrated that four novel markers, *SMC1B*, *RNF17*, *RBM46*, and *HORMAD1*, as well as representative markers commonly used to identify PGCs from gonads in avian species, such as *DAZL*, *DDX4*, *DND1*, and *PIWIL1*, can be applied to zebra finch (Figure 4A). Among the four novel markers, we verified experimentally that *RNF17* and *SMC1B* were expressed specifically in PGCs by performing germ cell specificity analysis of PGCs and gonadal stromal cells (GSCs). *RBM46* and *HORMAD1* were detected in both cell types and were not specific for PGCs (Figures 4B,C). Of the two markers verified by RT-PCR, *RNF17* was expressed not only in whole cells in reproductive tissues, but also in other tissues, whereas *SMC1B* was expressed specifically in reproductive tissues (Figure 4D). We confirmed experimentally that *SMC1B* expression in embryonic gonads of both sexes is maintained throughout development (Figure 4E and Supplementary Figure S7). Finally, we confirmed localization of the newly identified PGC marker *SMC1B* to germline cells by immunohistochemistry (Figure 4F) and *in situ* hybridization (Figure 4G) in HH stage 28 embryonic gonads. These results indicate that *SMC1B* gene, whose expression and function has not been elucidated in PGCs, may be a novel marker for identification of gonadal PGCs in zebra finch.

## Discussion

PGCs are the only cells that transmit genetic information to the next generation through gametogenesis; therefore, they are a focus of extensive basic and applied research. However, relatively little is known about the characteristics of PGCs, including their heterogeneity, in avian species compared with those in mammals and other species. Zebra finch is a vocal learning species within the clade Neoaves and has a fully sequenced genome; thus, it is used increasingly for neurobiological research. Accordingly, interest in zebra finch PGCs is increasing (Warren et al., 2010; Mello, 2014; Jung et al., 2019; Gessara et al., 2021). Here, we used an scRNA-seq approach to characterize zebra finch PGCs. We successfully identified three distinct PGC subpopulations at a single developmental stage, and detected a novel marker for zebra finch gonadal PGCs.

We obtained gene expression signatures for nine types of zebra finch gonadal cells (including PGCs and gonadal somatic cells) (Figures 1E,F). Some of the clusters identified as supporting cells, which are progenitor cells of Sertoli cells or granulosa cells, were shared with clusters assigned to steroidogenic cells, indicating that some of the progenitors of Leydig and theca cells are derived from a subset of supporting cells. Similarly, it has been reported that the steroidogenic lineage can be derived from supporting cells in chicken embryonic gonads based on the observation that some supporting cell marker (AMH)-positive cells simultaneously express the steroidogenic cell marker CYP17A1 (Estermann et al., 2020).

We found relatively high proportions of epithelial cells, interstitial cells, steroidogenic cells, and supporting cells, which contribute to regulating development by interacting with germ cells in the gonads. In particular, males had the highest frequency of supporting cells that differentiate into testicular Sertoli cells, and females had the highest frequency of epithelial cells that differentiate into ovarian cortex cells, indicating that the occupancy and importance of each cell type in the embryonic gonads has continuity in the development process (Estermann et al., 2020). Gonadal cell lineage specification during embryonic development may differ among vertebrates. Chicken supporting cells are derived from the mesenchyme population, unlike in mice (where supporting cells are derived from the coelomic epithelium) (Karl and Capel, 1998), and express *OSR1*, *WNT4*, *DMRT1*, and *PAX2* as representative marker genes. According to the differentiation of germ cells, levels of *OSR1* and *WNT4* increase, and those of *DMRT1* decrease, in female supporting cells, whereas levels of *OSR1* and *WNT4* decrease and *DMRT1* expression increases in male supporting cells, suggesting that supporting cells are involved in sex determination in chickens (Estermann et al., 2020). We speculate that the general underlying mechanism is similar to that in chickens because the gene expression patterns were similar (Supplementary Figure S8). This observation could be verified by analyzing germline cells in zebra finch at different developmental stages.

We demonstrated that cells annotated as PGCs based on expression of known germ cell markers can be clearly classified into three independent subtypes (Figure 3). Although c7 cells accounted for the largest fraction of PGCs, they were characterized by the fewest subtype-specific genes among the PGC subpopulations (Figures 2A,C). In the c7 PGC subtype, hemoglobin genes, *FOS*, and *JUN*, which are related to oxygen activity or reactive oxygen species (ROS), were specifically and highly upregulated. In several organisms, the modulation of ROS activity is crucial for maintaining cell fates in developmental contexts, and excessive oxygen free radicals can be associated with germ cell defects or death. Exogenously supplied oxygen free radicals induce testicular germ cell apoptosis in rats (Ikeda et al., 1999), increased ROS levels lead to PGC migration defects in fruit flies (Syal et al., 2020), and monkey ovarian germ cells exhibit an apoptotic fate due to oxidative stress following aging (Wang et al., 2020). In germ cells, autophagy can be induced by factors such as oxidative stress, hypoxia, nutrient depletion, and damaged organelles (Yadav et al., 2018). Among these, genes involved in *DDIT4*-induced autophagy via inhibition of mTOR and regulation of cellular stress responses were detected specifically in c7 PGCs. In our analysis, expression levels of *DDIT4* and *BECN1*, which induce autophagy, were high, and expression levels of *EEF2L* and *AKT1*, downstream of mTOR, were relatively low in c7 PGCs, suggesting cellular regulation by autophagy in this cell subset. The significantly enriched GO terms, such as “oxygen carrier activity” and “cellular response to reactive oxygen species,” for genes expressed specifically in c7 PGCs supports this claim. Furthermore, we found that the c7 PGC subtype with these general characteristics account for a large proportion (i.e., >50%) of the total PGCs. In early development, mouse germ cells are divided into “deciduous” germ cells, which are eliminated by apoptosis, and “actual” germ cells, which contribute to reproduction (Ueno et al., 2009). The presence of deciduous cells may be explained by the requirement for the germ cell–Sertoli cell interaction for Sertoli cell maturation and spermatogenesis (Weinbauer and Wessels, 1999; Ueno et al., 2009). We suspect that c7 PGCs play a similar role in zebra finch, assuming some similarity with the mechanisms underlying mouse germ cell activity.

The c12 PGC subpopulation, representing about 30% of the total PGCs, was characterized by a number of mitotic cell cycle-related genes and by relatively high expression levels of proliferation markers such as *ENSTGUG00000007339* (*BUB1*), *ENSTGUG00000021193* (*MKI67*), *PLK1*, and *E2F1*. In PGCs of several organisms, selection can also occur by proliferative heterogeneity rather than elimination. For example, two types of PGC founder cells, Z2 and Z3, in *C. elegans* exhibit proliferative asymmetry in response to nutritional cues (Fukuyama et al., 2006). In mice, PGC proliferation and mitosis are determined by the niche through which they migrate (Cantú et al., 2016). More proliferating germ cells can increase proportional expression within the gonads with successful division, leading to competition for niches or access to signaling factors that contribute to the differentiation program essential for germ cell development (Ueno et al., 2009; Nguyen et al., 2019). Thus, we suspect that the c12 PGC subtype with high proliferative ability could have a competitive advantage in changing developmental environments.

While expression levels of germness-related genes such as *ENSTGUG00000003145* (*DAZL*), *BOLL*, *ELAVL4*, and *ENSTGUG00000004779* (*PIWIL1*) were upregulated specifically in c12, we found that pluripotent genes such as *ENSTGUG00000013121* (*NANOG*), *SOX3*, *PRDM14*, and *TFAP2C* were detected selectively in c17 (Figure 3). In mice, PGCs that arrive first form a larger population than those of cells that arrive later, and this asynchronous migration affects proliferation and clonal expansion (Molyneaux et al., 2001; Cantú et al., 2016; Nguyen et al., 2019). Similarly, among subtypes of zebra finch PGCs, c12 PGCs, characterized by higher levels of proliferation and germness-related genes than those of other subtypes, are presumed to arrive first, and c17 PGCs, which specifically express genes related to migration and pluripotency, are presumed to be late-arriving in the gonads. The difference in arrival should be clarified in further studies.

Levels of pluripotency-related genes, including *ENSTGUG00000013121* (*NANOG*), were higher in c17 PGCs than in other PGC subtypes. Pluripotency-related genes control the transcription of germness-related genes in PGCs. In particular, *NANOG* is a key transcription factor required for formation of PGCs and the maintenance of early germ cells. *NANOG* regulates PGC-specific epigenetic programming and global histone methylation in mice (Leatherman and Jongens, 2003; Chambers et al., 2007; Choi et al., 2018). Avian PGCs follow a preformation mode that is formed autonomously by maternally-inherited germ plasm, unlike mouse PGCs that are induced by signals received from surrounding tissues during development (Murakami et al., 2016; Jung et al., 2018). However, the unique expression of the pluripotency-related genes in c17 PGCs suggests that epigenetic events influence avian PGCs during embryonic development. Additionally, we found several genes related to cellular localization, morphology, and signaling pathways in c17 PGCs. The location and morphological characteristics of c17 PGCs are presumed to reflect differences in migration, and the differential expression of many genes involved in signaling pathways identified in c17 PGCs suggests that signaling mechanisms differ among PGC subpopulations (Supplementary Table S3). Even synthesizing all available evidence to date, it has not been possible to define c17 PGCs as a specific functional group. We expect our results regarding the heterogeneity of these PGCs to contribute to detailed analyses of c17 PGC-specific functions.

Finally, we identified *SMC1B* as a novel marker for zebra finch PGCs in embryonic gonads by screening for a common PGC-specific gene in the three subpopulations (Figure 4). *SMC1B*, one of the components of the meiotic cohesin complex, is a meiotic germ cell marker. It has gonad specificity and is highly conserved across a variety of species (Nicholls et al., 2019). *SMC1B* is required for meiotic chromosome dynamics, sister chromatid cohesion, and DNA recombination; indeed, *SMC1B*-deficient spermatocytes showing abnormalities in chromosome structure also show high rates of apoptosis during pachytene in mice (Revenkova et al., 2004; Nicholls et al., 2019; Zhao et al., 2020; Ge et al., 2021). The role of *SMC1B* is not limited to germ cells; it is also involved in the DNA damage response by interactions with mitotic cohesin in somatic cells in mice (Mannini et al., 2015). The exact role of *SMC1B* in undifferentiated PGCs is unknown; however, based on our findings, it is expected to be revealed in the near future.

Studies of the heterogeneity of avian PGCs and cellular level selective events are still at an early stage, and analyses of other avian species are needed. To the best of our knowledge, this study is the first to report scRNA-seq analysis of zebra finch; therefore, it has many practical limitations. First, it was difficult to access each independent PGC subpopulation experimentally owing to a lack of sufficient experimental materials such as antibodies (compared with those available for other laboratory animals). As an alternative, multiplex *in situ* hybridization was attempted, but there were still practical difficulties in finding specific probe sequences that can distinguish them and establishing multiple *in situ* hybridization conditions in zebra finches. Nevertheless, our results contribute to germ cell research by providing the first evidence for heterogeneity in avian PGCs. We believe that the current methodological limitations can be overcome by gene-editing technology and marker development. Second, the effects of sexual differences could not be investigated in a statistical framework owing to the absence of biological replicates. Most scRNA-seq experiments do not yet consider multiple biological replicates owing to their high cost (Haque et al., 2017; Natarajan et al., 2019; Zhang et al., 2019); however, as prices decrease, more precise statistical approaches, such as the current bulk RNA-seq technology, will be possible. However, our study addresses the lack of research on cell-specific expression patterns in zebra finch. The results provide a basis for further, more detailed analyses of avian PGCs.

The results of our scRNA-seq analysis provide a deeper understanding of avian PGCs by revealing for the first time the heterogeneity and intrinsic changes of gonadal PGCs, which have hitherto been considered a single population in birds. We believe that the single-cell level profiling results will be useful for future investigations of selection on variation in avian germ cells during embryonic development.

## Data Availability

The scRNA-seq data generated here are available in the Gene Expression Omnibus database under accession code GSE177478.
