# Supplementary material for: Single-Cell RNA Sequencing Revealed the Heterogeneity of Gonadal Primordial Germ Cells in Zebra Finch (Taeniopygia guttata)
Source: Front Cell Dev Biol. 2021 Dec 9;9:791335. doi: 10.3389/fcell.2021.791335 (PMC8695979; doi:10.3389/fcell.2021.791335)
Supplement: Supplementary file 3 [file DataSheet1.pdf]

Supplementary Material

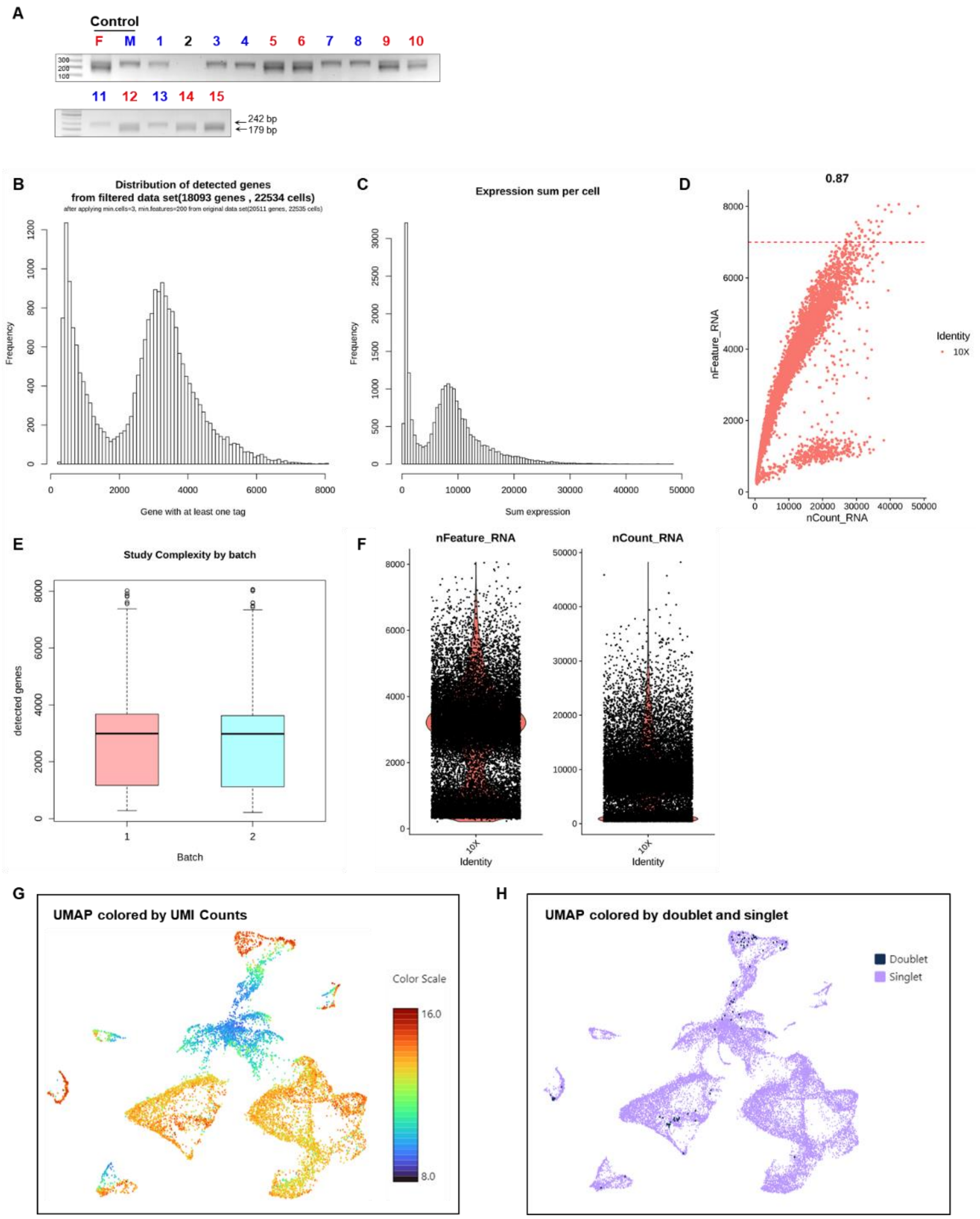

Supplementary Figure 1. Single-cell RNA sequencing data pre-processing and quality check. (A) PCR-based sex identification of zebra finch embryos. Genomic DNA was extracted from embryonic blood taken from 3-days eggs. Samples with known sexes were used as positive controls. A total of 15 embryo samples were analyzed. The numbers at the top of gel images are randomly assigned to the embryos. Males are indicated in blue and females are indicated in red letters. Seven embryos were identified as male, seven embryos were female, and one embryo was unknown. Seven sex-identified embryos were selected from each sex for scRNA-seq analysis. (B) The histogram shows the frequency of expression sum per cell. Raw data set is non-normalized data on 20,511

genes, 22,535 cells. (C) The histogram shows the frequency of cells containing genes with at least one UMI after filtering out cells/features from original data set (First filtering criteria : keep all genes expressed in >= 3 cells. keep all cells with at least 200 detected genes). First filtered data is a feature-barcode matrix with 18,093 genes, 22,534 cells after filtering by min. cells=3, min.features=200. (D) The scatter plots represent relationships of genes-molecule counts for each cell. nFeatures counts any genes with at least 1 UMI count. nCount is the number of UMI per cell. (E) The bar plots show study complexity by batch. Batch 1 indicate male and batch 2 indicate female. (F) The violin plots represent the distribution of nGene/nUMI for each cell. (G) UMAP plot colored by UMI counts. (H) UMAP plot colored by doublet and singlet. Doublet was detected by Scrublet.

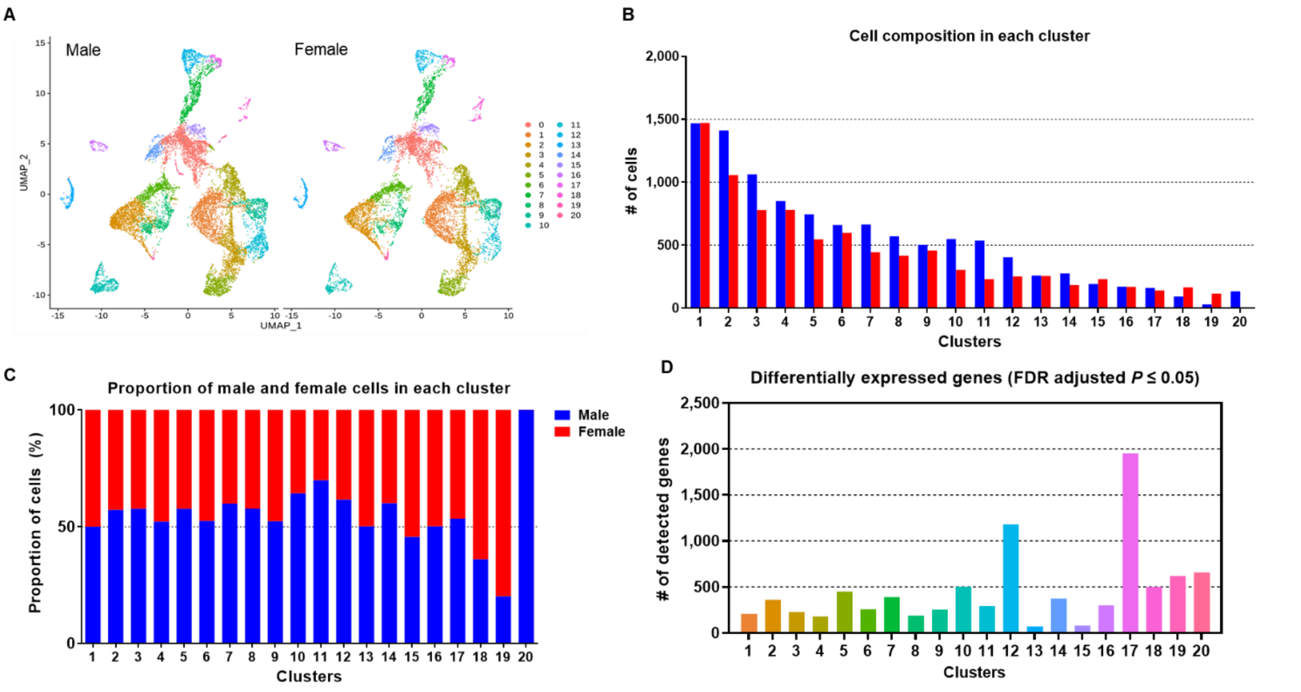

Supplementary Figure 2. Single-cell RNA sequencing cluster composition analysis. (A) UMAP clustering by gender. Males and females showed 20 and 19 distinct clusters, respectively (c20 only existed in male). (B) Number of cells assigned to each cluster in both sexes. (C) Proportions of male and female cells in each cluster. (D) Number of differentially expressed genes (DEGs) in each cluster based on aggregated UMAP clusters.

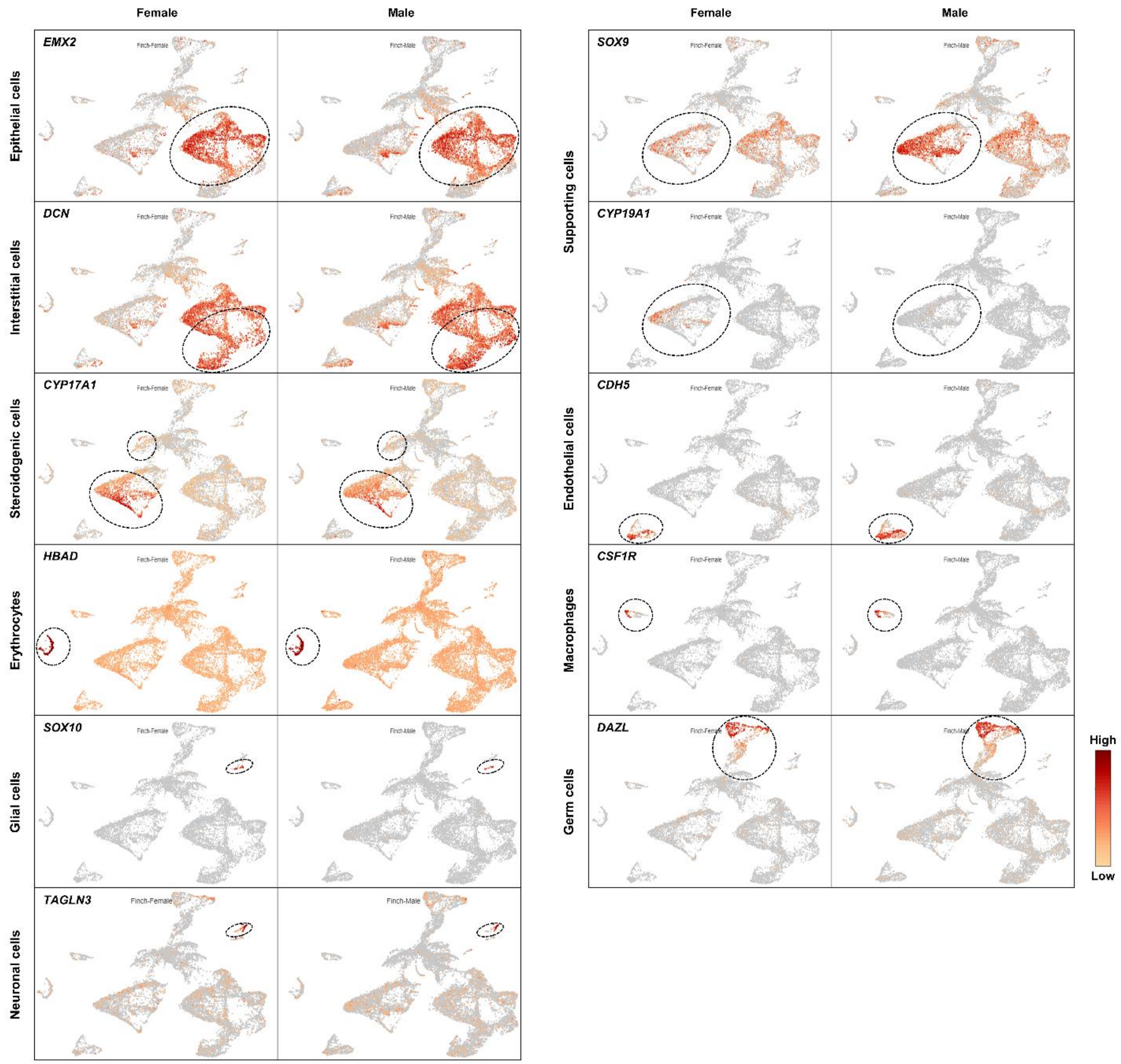

Supplementary Figure 3. Assignment of gonadal cell types in each sex based on the gene expression of known germ cell markers. UMAP plots of representative marker genes for each cell type are shown. For supporting cells, *CYP19A1* was used as female marker and *SOX9* was used as male marker. For other cell types, the same markers were used for both sexes.

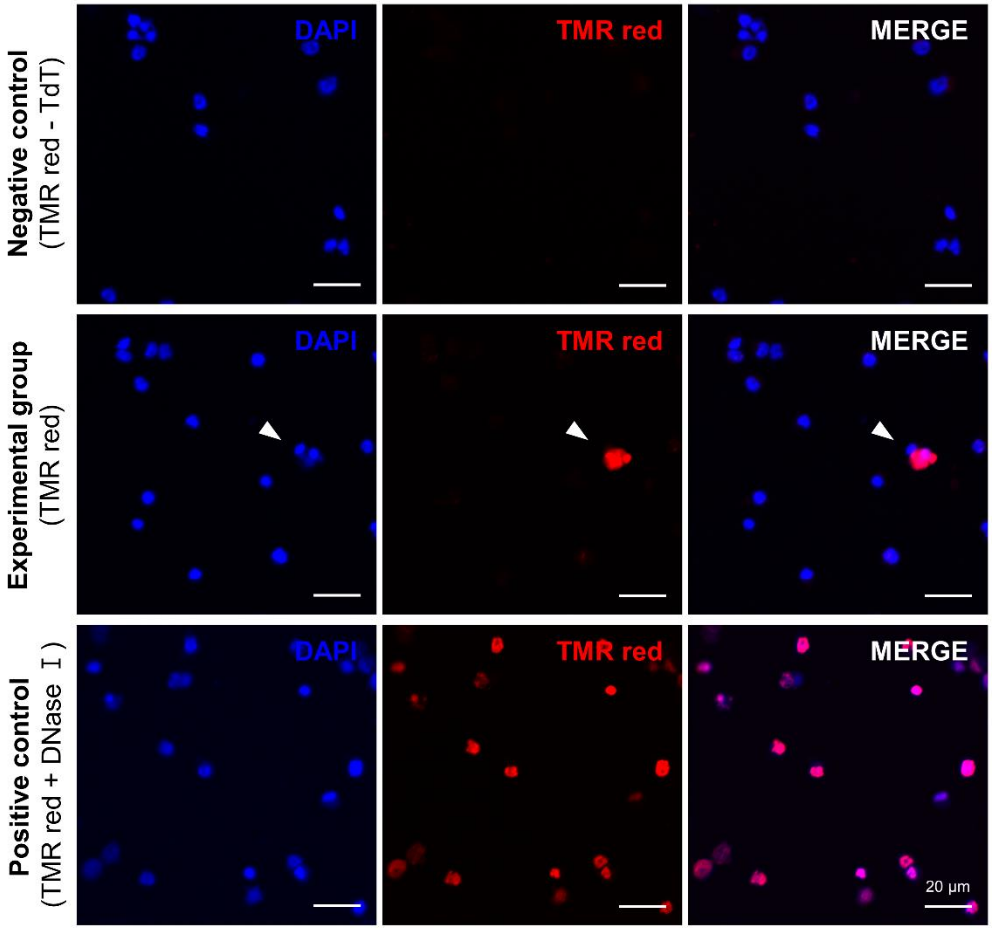

Supplementary Figure 4. Detection of the apoptotic cells in zebra finch embryonic gonadal cells at HH stage 28. Whole gonadal cells were treated with the TUNEL reaction mixture (TMR red labeling). Negative control was treated with the mixture without terminal deoxynucleotidyl transferee (TdT). Cells induced DNA damage by treatment with DNaseI were used as a positive control. In experimental group, apoptotic cells were detected (indicated by white arrows). Nuclei were stained by DAPI.

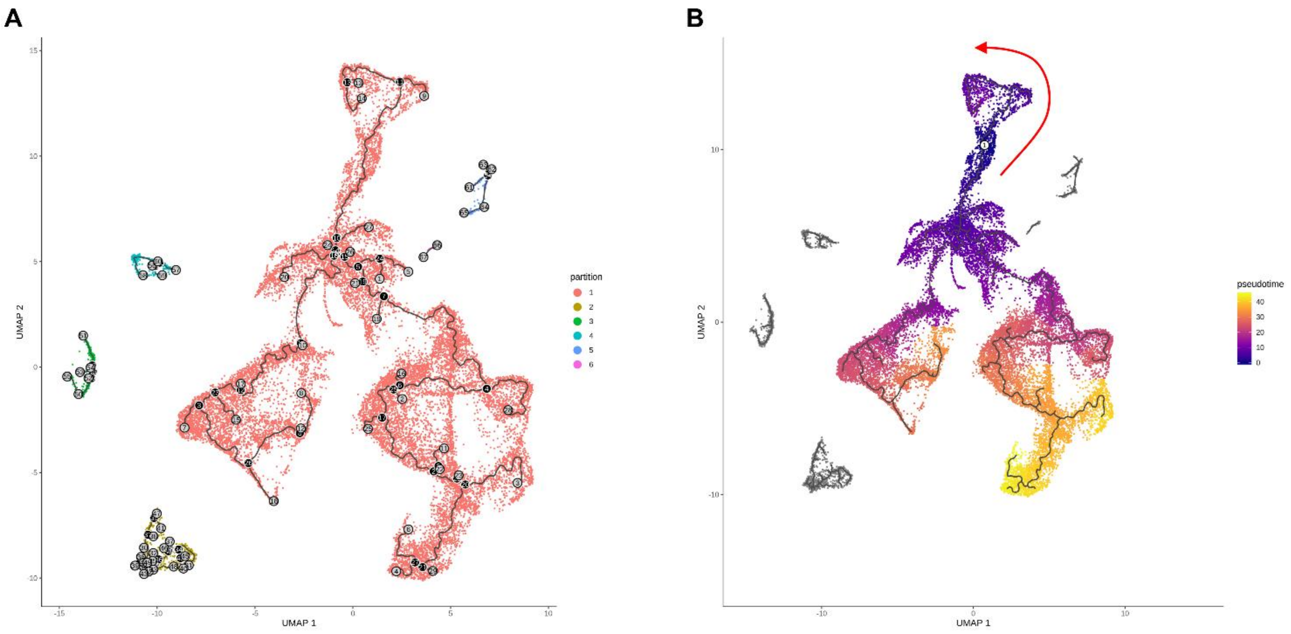

Supplementary Figure 5. Pseudotime trajectory analysis of PGC subpopulations. (A) Trajectory graph by partition for the zebra finch whole embryonic gonadal cells. (B) Trajectory graph with pseudotime. Red arrow indicates continuous pseudotime trajectory for the three PGC subpopulations.

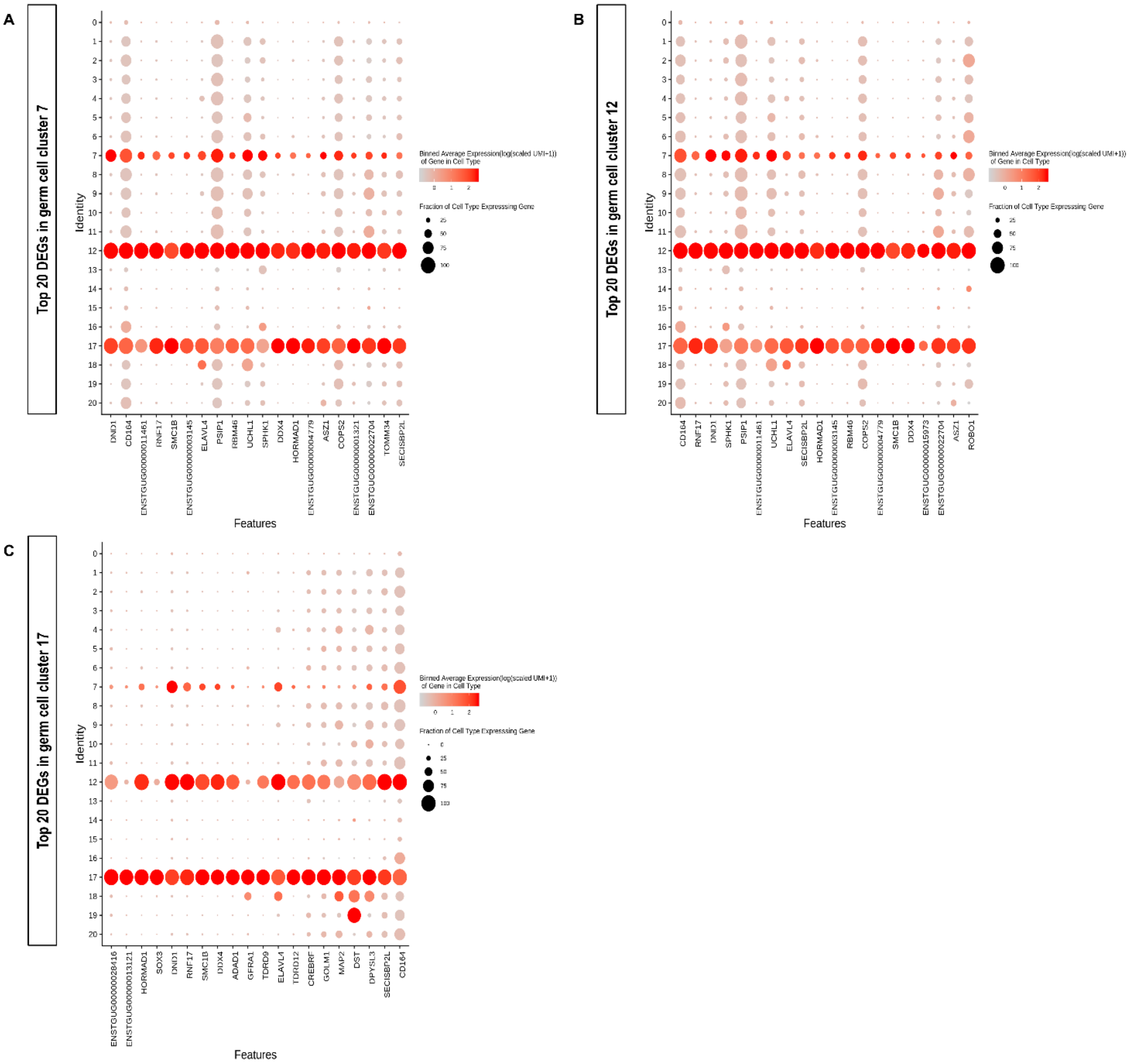

Supplementary Figure 6. Top 20 genes with cell type-specific expression patterns in the three PGC subtypes. By examining the top 20 genes in each PGC subtype, it was confirmed that each cluster showed a specific gene expression pattern, suggesting that the genes can be used as gonadal PGC-specific markers in zebra finch.

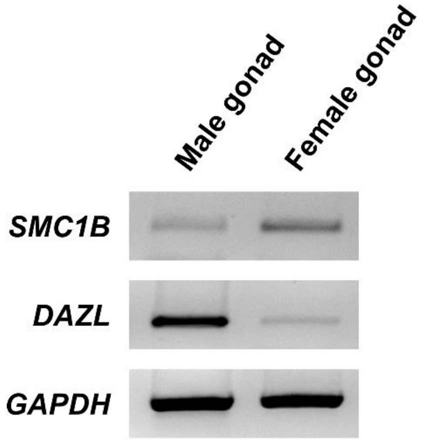

Supplementary Figure 7. RT-PCR analysis of *SMC1B* gene in embryonic gonads prior to HH stage 28. Male and female zebra finch embryonic gonads at HH stage 26 were used for analysis.

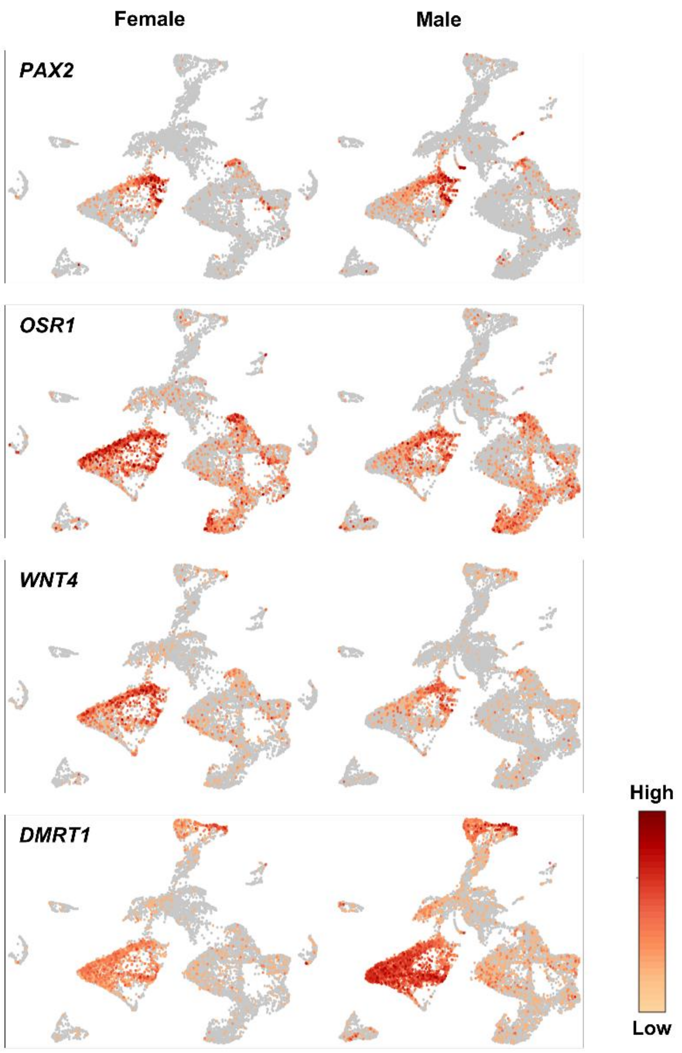

Supplementary Figure 8. Expression of avian supporting cell-specific genes in zebra finch gonadal cells. UMAP plots of representative marker genes in chicken supporting cells are shown. All four genes were specifically expressed in the supporting cell cluster in both sexes.
